# Mechanical and Enzymatic Procedures to Isolate the Stromal Vascular Fraction From Adipose Tissue: Preliminary Results

**DOI:** 10.3389/fcell.2019.00088

**Published:** 2019-06-07

**Authors:** Letizia Senesi, Francesco De Francesco, Luca Farinelli, Sandra Manzotti, Giulio Gagliardi, Giuseppe Francesco Papalia, Michele Riccio, Antonio Gigante

**Affiliations:** ^1^Clinical Orthopaedics, Department of Clinical and Molecular Science, Università Politecnica delle Marche, Ancona, Italy; ^2^Department of Plastic Reconstructive Surgery and Hand Surgery, Azienda Ospedaliero Universitaria Ospedali Riuniti, Ancona, Italy; ^3^Regenerative Surgery, Research and Training Center, Lipofilling Academy, Ancona, Italy

**Keywords:** adipose stem cells, stromal vascular fraction, adipose tissue, osteoarthritis, regenerative medicine

## Abstract

Adipose-derived MSCs (ASCs) and stromal vascular fraction (SVF) play an important role in regenerative medicine and in the treatment of osteoarthritis. ASCs extracted from lipoaspirates are a valuable cell source due to their abundance and accessibility. ASCs are retrieved from the aqueous fraction of the digested lipoaspirate. The aqueous fraction is known as SVF and includes, ASCs, endothelial precursor cells (EPCs), endothelial cells (ECs), macrophages, smooth muscle cells, lymphocytes, pericytes, as well as pre-adipocytes. To date, two types of techniques to isolate SVF have been proposed: enzymatic and mechanical. The enzymatic method is particularly indicated in SVF isolation since it disrupts the extracellular matrix (ECM) and the binding of adipocytes and other cells but is restricted by regulatory issues related to enzymatic procedures, especially within the European Community. Thus, making the search for alternative mechanical methods imperative. This study assesses the SVF harvested from subcutaneous abdominal fat via two different mechanical procedures and the standard enzymatic method to evaluate their eligibility in a clinical context. In particular, we analyze cell viability (at 0 and after 72 h) as well as the expression of cluster differentiation (CD) for each sample and the differentiation in adipocytic, chondrocytic, osteocytic linage. The mechanical procedures yielded no significant difference in cell viability and cluster differentiation pattern expression, even if enzymatic procedure still remain the “gold standard.” We retain that clinical efficacy in treating ostheoarthrosis with SVF administration is probably related to his anti-inflammatory and immunoregulatory effect, rather than the ability to differentiate in specific cell lineage. However, further studies are required to support and improve our findings.

## Introduction

Osteoarthritis (OA) is a well-established form of rheumatic disease, denoted by the degeneration of articular cartilage, synovial inflammation and changes in the subchondral bone. The pathophysiology of OA is still unclear, but it is known that the degradation of cartilage is mainly due to the prevalence of catabolic processes as well as reduced cartilage cellularity with aging (Martin and Buckwalter, 2001). Currently, regenerative therapies for OA are scarce and are not effective inrelation to the gradual degeneration of joint tissues within a clinical context and mainly focuses on reducing pain and improving articular function. Therefore, non-steroidal anti-inflammatory drugs, infiltrative therapies and the total joint replacement represent the main treatments in clinical practice (Conaghan et al., 2008). However, considering that OA is characterized by degeneration and failure in repairing cartilage due to lack of blood supply, it is then, relevant to consider mesenchymal stem cells (MCs) in OA management (Coleman et al., 2010).

Friedenstein first defined MSCs, illustrating a subpopulation of cells that contained osteogenic potential within the bone marrow stromal (Friedenstein et al., 1966). Nowadays, MSCs are commonly isolated from adipose tissue, the synovium, periosteum, dermis, bone trabeculae, infrapatellar fat pad and muscle with similar phenotypes but differing proliferative and differentiative abilities (Lodi et al., 2011). MSCs have undoubtedly a critical role in homeostasis and tissue repair with the ability to differentiate into all connective lineages of tissue depending on the stimuli provided (Mobasheri et al., 2014).

Adipose-derived MSCs (ASCs) from lipoaspirates are an effective cell source due to their abundance accessibility and ability to differentiate in several cellular lineage.

Some studies demonstrated for the first time that ASCs positive for pericytic markers, including NG2, when loaded on a crosslinked hyaluronic acid scaffold and grafted into nude mice was able to differentiate in muscle tissue (Desiderio et al., 2013). Another study reveal that that hyaluronic acid potentiate ASCs differentiation of resident fat cell of the face preserving both morphology and viability (Stellavato et al., 2017). It was also largely used in breast lipofilling even caution should be paid, because it has been demonstrated that hASCs may enenced tumor angiogenesis (Paino et al., 2017).

The increasing interest about using ASCs in osteoarticular disorders are enforced because different studies revealed that ASCs had potentiality to differentiate into bone tissue (Marshall et al., 2019) and into cartilage (Zuk et al., 2001). Current research has favorably described different uses of these cells for infiltration or for scaffold implants employing different biomaterials, growth factor, hyaluronic acid or platelet rich-plasma (Filardo et al., 2013). The most effective strategy has, indeed yet to be defined and will not be discussed herein. ASCs are isolated by the aqueous fraction derived from the digestion of lipoaspirates. This aqueous fraction constituted by ASCs is known as the stromal vascular fraction (SVF) containing endothelial precursor cells (EPCs), endothelial cells (ECs), macrophages, smooth muscle cells, lymphocytes, pericytes, and pre-adipocytes (Bora and Majumdar, 2017). The clinical application of ASCs and SVF is steadily increasing along with a need for steadfast information regarding adequacy and cost of automated and manual appliances to isolate SVF from adipose tissue. Two techniques have currently been proposed: enzymatic and mechanical methods with evidence of a more efficient enzymatic procedure in the separation of SVF which disarrays the extracellular matrix (ECM) and binds adipocytes from other cells (Zuk et al., 2001). However, in view of the Good Manufacturing Practice regulations regarding “cell manufacturing” (Raposio and Ciliberti, 2017; Agostini et al., 2018), the extensive use and manipulation of stem cells are not applicable according to the European Parliament and Council (EC regulation no. 1394/2007) in relation to the minimal manipulation (De Francesco et al., 2018). An alternative mechanical procedure as compared to the enzymatic method is therefore imperative to isolate SVF (Aronowitz et al., 2015). In this study, we analyze the SVF harvested from subcutaneous abdominal fat from five patients derived from two different mechanical methods and the most common enzymatic method. In particular, we focus on SVF isolated from the various methods, cell viability (at 0 and after 72 h) and the expression in clusters of differentiation (CD) and the ability to differentiate in adipocytic, chondrocytic, osteocytic lineage.

## Materials and Methods

### Patients and Tissues

Between February 2018 and June 2018, five patients (three women and two men) underwent liposuction and lipofilling procedures for esthetic purposes with ages ranging between 45 to 65 years (mean 54.3 ± 10). Inclusion and exclusion criteria are summarized in Table 1. Patients provided consent before surgery, in line with the ethical guidelines set by the review board for human studies of the “Ospedali Riuniti” Hospital, Ancona, Italy (Micro-adipose graft_01, 18 May 2017). The liposuction procedure was performed according to the BEAULI protocol, described by Ueberreiter et al. (2013). A pulsating water jet was used for infiltration and simultaneous aspiration. The infiltration ranges were set to 1–3 from 90 mL/min ± 15% to 130 mL/min ± 15%. Klein’s tumescence solution was pre-heated at 37–38°C. The sharp-tipped cannulas measured 22 mm in external diameter. We proceeded with aspiration promptly after the first infiltration. For each patient, we collected 30 mL of lipoaspirate tissue equally divided (10 mL) for each procedure.

**Table 1:** We reported the exclusion and inclusion criteria of patients’ characteristics.

Inclusion criteria	Exclusion criteria
Age < 65 aa	Diabetes mellitus type I and II
Body Mass Index >25 kg/m^2^ < 30 kg/m^2^	Cardiovascular or neurologic disorders
	Patients in chronic drug therapy
	Smoke
	Previous abdominal surgery (laparotomy)


### Enzymatic Isolation of SVF

One portion of lipoaspirates from each patient was digested with collagenase according to the preceding protocol (Filardo et al., 2013). The lipoaspirates were briefly digested at 37°C in DMEM with 0.25% weight per volume percent (w/v) collagenase type I (Sigma-Aldrich, St. Louis, MO, United States) and 1% fetal bovine serum (FBS) for 180 min at 37°C. Following digestion, we filtered the resulting suspension through a sterile 100 μm nylon mesh to remove undigested parts and centrifuged the remaining suspension at 1200 *g* for 10 min, to extract a high-density pellet, composed of the SVF. The SVF thus obtained was re-suspended in 10 mL DMEM supplemented with 10% Foetal Bovine Serum (FBS) and 1% penicillin-streptomycin (all Gibco^®^, Thermo Fisher Scientific, Paisley, United Kingdom), seeded in 96-well plates (100 μl/well) and were stored in a humidified incubator at 37°C with 5% CO_2_ to assess viability. The remaining part was seeded in 60 mm petri dishes and used for flow cytometric characterization.

### Non-enzymatic Methods

In this study we evaluated two mechanical technologies: Rigenera^®^ (Human Brain Wave HBW, Turin, Italy), Lipogems^®^ ortho kit (Lipogems International SpA, Milan, Italy). Two portions of the harvested fat of each patient was inserted into each device, according to each manufacturer’s instructions (Bianchi et al., 2013; Filardo et al., 2013; Domenis et al., 2015). The SVF obtained with each method, as for the SVF obtained by enzymatic digestion, was brought to the volume of 10 mL with DMEM supplemented with 10% FBS and 1% penicillin–streptomycin (all Gibco^®^, Thermo Fisher Scientific, Paisley, United Kingdom). Cells was seeded in 96-well plates (100 μl/well) and were stored in a humidified incubator at 37°C with 5% CO_2_ to assess viability. The remaining part was seeded in 60 mm petri dishes and used for flow cytometric characterization.

### Evaluation of Cell Viability

The viability of cells was evaluated through PrestoBlue test (Life Technologies Corporation, OR, United States) following each manufacturer’s protocol. Hundred microliters of cell suspension obtained from each processing method was seeded in a 96-well micro titer plate. Subsequently, 10 μl of PrestoBlue reagent was added. The cell viability alterations were analyzed via absorbance spectroscopy and we reported the absorbance 570 nm after 10 min incubation at 37°C through multi-mode microplate readers (Tecan Trading AG, Switzerland). Cell viability was evaluated at T0 and T (72h) and was performed in triplicate in every sample for each method.

### Flow Cytometry Analysis

At confluence, cells derived from each technique were detached with trypsin-EDTA (Gibco^®^, Thermo Fisher Scientific, Paisley, United Kingdom). Cells were washed with PBS, about 2 × 10^5^ were placed in 5 mL round-bottom polystyrene tubes and then stained for 30 min at 4°C using fluorescent conjugated antibodies. The following monoclonal antibodies were investigated: anti-CD34 PE and anti-CD45 PE (both Diaclone, Besançon, France), anti-CD90 FITC (StemCell Technologies, Vancouver, BC, Canada), anti-CD73 PE (BD Biosciences Pharmingen, San Jose, CA, United States), anti-CD235a (Miltenyi Biotec, Germany) and anti-CD31 1:50 (Dako Agilent, Santa Clara, CA, United States). After 45 min, cells were washed and then stained with goat anti-mouse IgG FITC secondary antibody 1:500 (Invitrogen, Thermo Fisher Scientific). After immunostaining, the cells were rinsed in PBS, re-suspended in 0.5 mL of FACSFlow and examined by FACSCalibur flow cytometry system (Becton Dickinson, CA, United States). We used isotypic mouse IgG1 as control for FITC and PE-coupled antibodies, and secondary antibody only for indirect immunostaining. Data were collected and analyzed via CellQuest software (BD Biosciences, San Jose, CA, United States).

### Differentiation

One sample was chosen randomly and was divided in three aliquote to perform phenotypical differentiation of enzymatic and mechanical processed SVF. For each type of treatment, the material was divided as follows: two thirds used for chondrogenic differentiation and a third for adipogenic and osteogenic differentiation.

Cells were cultured 10 mL DMEM supplemented with 10% Foetal Bovine Serum (FBS) and 1% penicillin-streptomycin (all Gibco^®^, Thermo Fisher Scientific, Paisley, United Kingdom), until confluency and medium was then substituted. The StemPro^®^ Chondrogenesis Differentiation Kit, StemPro^®^ Adipogenesis Differentiation Kit, and StemPro^®^ Osteogenesis Differentiation Kit, were used (all Gibco^®^, Thermo Fisher Scientific, Paisley, United Kingdom). The medium was changed twice a week. For osteogenic and adipogenic differentiation we used 35 mm petri. Indeed, for chondrogenic differentiation 15 mL centrifuge tubes were used, according to our experience.

After 14 days the cells obtained, cultivated in osteogenic medium were fixed with 4% formaldehyde and stained for ALP (alkaline phosphatase). After 21 days the cells obtained, cultivated in osteogenic medium were fixed with 4% formaldehyde and stained with Von Kossa and Alizarin red to highlight mineral deposition. The cells cultivated in medium with adipogenic medium were fixed in methanol and stained with Oil red O to highlight lipid droplets.

After 28 days the pellets formed in the centrifuge tubes with chondrogenic medium were fixed with 4% formaldehyde and processed for paraffin embedding. Five micrometers sections were stained with Safranin O and Alcian blue pH1 to highlight the presence of sulfurated acid proteoglycans. The Alcian blue pH 1 solution was purchased by Bio-Optica (Milan, Italy), the other staining solutions were prepared in the laboratory with Sigma-Aldrich products (Milan, Italy).

### Statistical Analysis

Population characteristics were defined using a mean ± standard deviation or median and range for the continuous variables and percentages related to the categorical variables. Data were assessed for normal distribution using the Kolmogorov–Smirnov test. Statistical significance was set at *p* < 0.001. We used the Paired *t*-test to compare the continuous variables between the two groups and the result was considered statistically significant when *p* < 0.005.

## Results

### Cell Viability Assay

**FIGURE 1:**
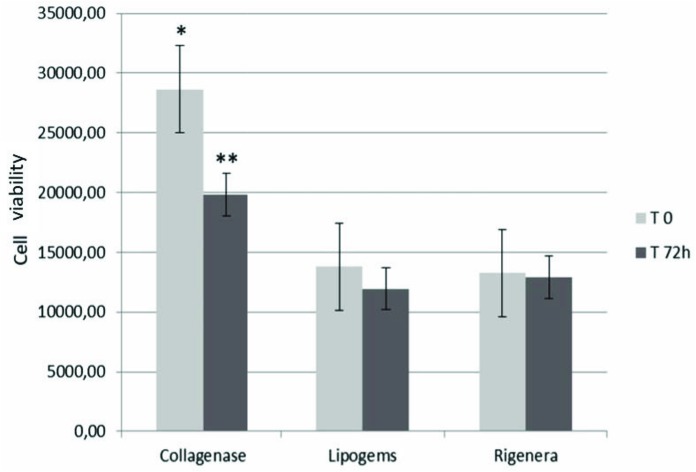
Cell viability values (average of the samples) at T0 and T72h. Data marked with ^∗^ are statistically significant. Cell viability of SVF obtained by collagenase was significantly higher than those of mechanical methods (^∗^). However, the enzymatic procedure has been the only methods characterized by a significant decrease in cell viability at T72h^∗∗^. Cell medium was changed twice weekly in DMEM supplemented with 10% Foetal Bovine Serum (FBS) and 1% penicillin-streptomycin (all Gibco^®^, Thermo Fisher Scientific, Paisley, United Kingdom), seeded in 96-well plates (100 μl/well) and were stored in a humidified incubator at 37°C with 5% CO_2_ to assess viability.

Cell viability at T0 and T72h for each method is summarized in Figure 1. We reported the average cell viability obtained from each method. The cell count was statistically higher at T0 for enzymatic technique of isolation in relation to the other mechanical methods (Collagenase: 28634^∗^, Lipogems^®^:13782 Rigenera^®^:13262). Moreover, at T72h cell viability was statistically lower in the Collagenase method only compared to T0 (19797), while the difference was statistically insignificant in the other group (Lipogems^®^:11950, Rigenera^®^:12932).

### Cluster of Differentiation Analysis

**FIGURE 2:**
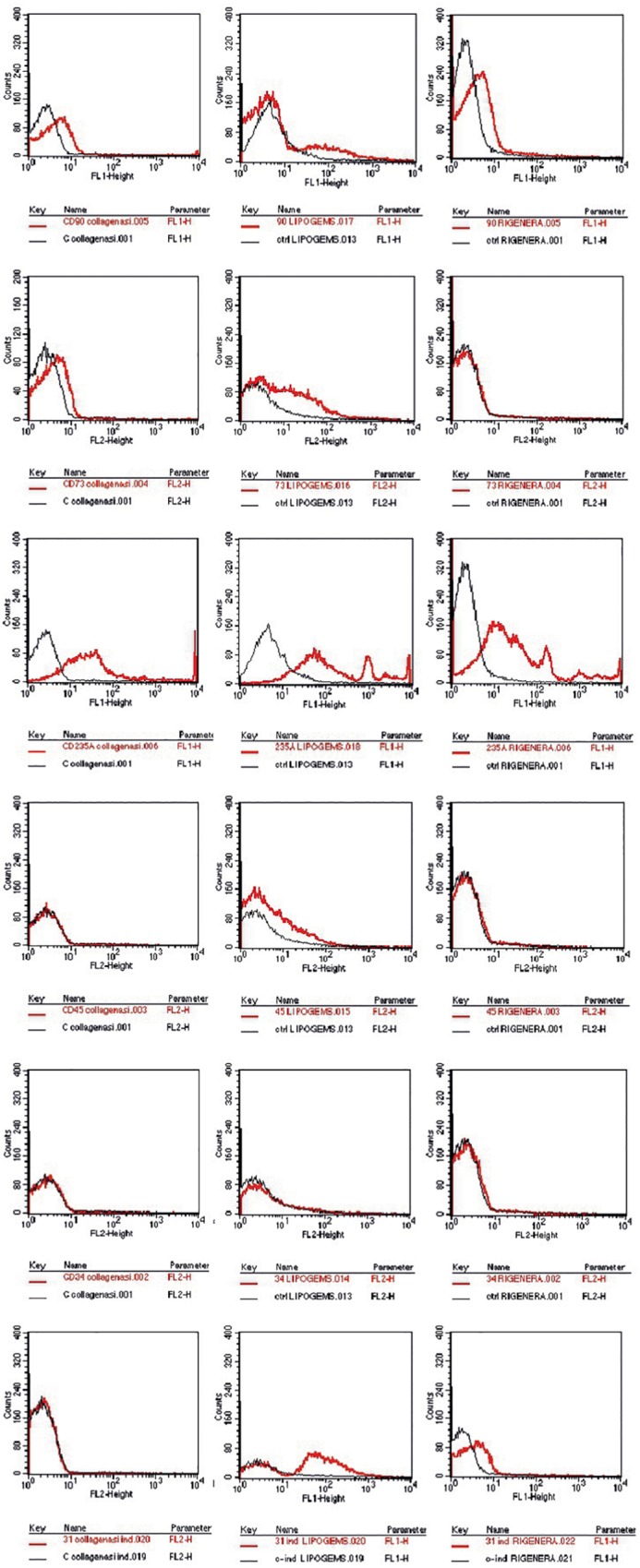
CD expression marker of one sample chosen randomly, showed as exemplification. In the first column the enzymatic technique reveals CD90, 73, 235a+, and CD45, 34, 31–. For the mechanical techniques, in the second Lipogems system CD90, 73, 235a, 45, 31+, and CD34–. Rigenera system in the last column CD90+, 73–, 235a+, and CD45–, 34–, 31+.

**FIGURE 3:**
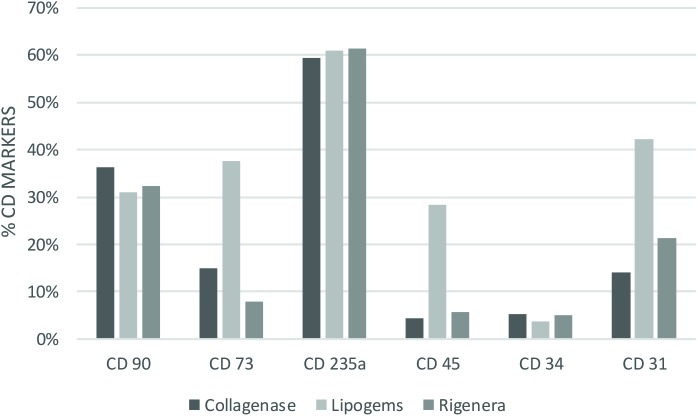
Percentage of staining CD cell (as average of the samples) for each method and technique analyzed. Collagenase: CD90 36% (+), CD73 15% (–), CD235a 59% (+), CD45 4% (–) CD34 5% (–) CD31 14% (–). Lipogems: CD90 31% (+), CD73 38% (+), CD235a 61% (+), CD45 28% (+) CD34 4% (–) CD31 42% (+). Rigenera: CD90 32% (+), CD73 8% (–), CD235a 61% (+), CD45 6% (–) CD34 5% (–) CD31 21% (+).

Cytofluorimetric analysis of one sample processed by the enzymatic method and mechanical methods is reported below. Non-overlapping curves indicated positive staining, and overlapping curves indicated a negative value (Figure 2). The percentage of staining CD markers are reported for each method (as an average of samples) in Figure 3. Data were considered positive or negative according to Cell Quest software for statistical analysis and D/s(n) value.

### Phenotypic Differentiation

After 14 days the cells obtained through Collagenase cultivated in osteogenic medium were stained for ALP (alkaline phosphatase). There were no adherent cells in petri dishes with Rigenera^®^ and Lipogems^®^ treatment.

After 21 days the cells obtained through Collagenase, cultivated in osteogenic medium were stained with Von Kossa and Alizarin red to highlight mineral deposition. There were no adherent cells in petri dishes with Rigenera^®^ and Lipogems^®^ treatment.

The cells obtained from the treatment with collagenase are cultivated in medium with adipogenic medium. There were no adherent cells in petri dishes with Lipogems^®^, and Rigenera^®^ treatment.

**FIGURE 4:**
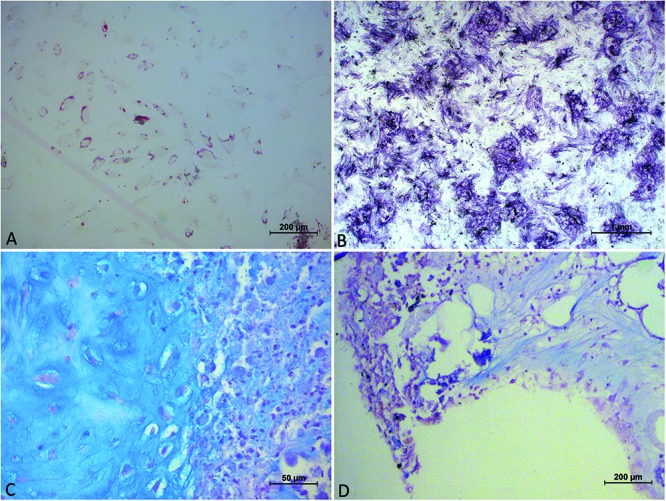
Image showing panel **(A)** cells, obtained from collagenase digestion, in adipogenic medium, exhibiting the morphology of multi-vacuolar adipocytes (arrows; Scale bar: 200 μm); **(B)** cells, obtained from collagenase digestion, in osteogenic medium. Formation of mineralized matrix was detected by Von Kossa staining (arrows; Scale bar: 1 mm); **(C)** cells, obtained from collagenase digestion, in chondrogenic medium demonstrated by Alcian Blu staining; **(D)** cells, obtained from Rigenera digestion, in chondrogenic medium demonstrated by Alcian Blu staining.

After 28 days the pellets formed in the centrifuge tubes with chondrogenic medium for Collagenase and Rigenera^®^ were fixed. Lipogems^®^, did not show in our opinion capability of differentiation. Rigenera shows the ability to differentiate in chondrocytic lineage (Figure 4). Osteocytic, adipocytic, and chondrocytic differentiation was achieved only in enzymatic technique.

## Discussion

To date, autologous adipose SVF injection represents a promising, minimally invasive, non-surgical alternative in the field of orthopedics for the treatment of musculoskeletal disorders. Although various pre-clinical and clinical studies have been conducted to establish the potential role of ASCs and SVF to treat OA and/or cartilage lesions, a unanimous conclusion is still lacking due to inadequate standardization (Bora and Majumdar, 2017). Enzymatic methods produce cell populations considered “more than minimally manipulated,” by the Food and Drug Administration and thus strictly regulated as a therapy. For these reasons, non-enzymatic methods were defined as “minimally manipulated” and were developed to circumvent the extensive regulations (Raposio and Ciliberti, 2017; Agostini et al., 2018; De Francesco et al., 2018). According to our findings, collagenase-based digestion seemed to be more effective in terms of cell viability at T0 and T72h compared to mechanical techniques. As reported in literature (Condé-Green et al., 2014) non-enzymatic methods revealed lower cell viability compared to enzymatic methods. However, differences between T0 and T72h in cell viability, were significantly lower in the enzymatic technique only, compared to other groups, in which an almost steady state of viability was maintained. No difference in cell viability between mechanical methods in both time points was reported.

Our study is inconclusive regarding the characterization of cluster differentiation. It is noteworthy that SVF comprises various cells such as macrophages, numerous blood cells, pericytes, fibroblasts, smooth muscle cells, vascular endothelial progenitors and ASCs. ASCs are known to be positive for classical MSC markers such as CD34, CD73, and CD90 but are not so for the pan-leucopoietic lineage marker CD45. In addition, progenitors of hematopoietic and endothelial lineage are expressed in CD34 whereas endothelial cells are positive for CD31 (Bora and Majumdar, 2017). CD235a is unexpressed in lymphoid or granulocytic progenitor cells and thus an efficient marker to detect the erythroid cell lineage. The composition of the cell populations retrieved via enzymatic methods are known to contain a higher frequency of peripheral blood. As regards the ASCs, we reported a strong positivity of CD90 and CD73, but a negativity of CD34. Recent investigations indicate that CD34 is expressed in tissue-resident MSCs, and its negativity is a consequence of cell culturing (Lin et al., 2012). On the other hand, Dominici et al. (2006) stated that ASCs were positive for MSC markers CD90, CD105, and CD73, and negative for CD45, CD11b, HLA-DR, CD34, and CD19 according to the international criteria which defines multipotent MSCs. For these reasons, we hypothesize that ASCs are present in SVF albeit at a substantially lower rate than in red blood cells.

Cluster differentiation expression in Collagenase technique showed the expression of CD90, CD235a and negativity for CD34, CD45, CD73, and CD31. Negativity for CD73 is borderline. CD expression in the mechanical methods Rigenera^®^ suggested that the SVF obtained was characterized by a substantial proportion of red blood and endothelial cells. Indeed, they expressed CD31, CD90 with a negativity for CD34, CD45, CD73 (Ades et al., 1980). Concerning Lipogems^®^, we observed a strong positivity for CD235a, CD31, CD90, CD73, CD45 with a negativity of CD34. Our studies revealed a collection of a significant amount of red blood cells. Furthermore, as stated above, the positivity of CD73 and CD90 suggests the presence of ASCs. The main limitations of our study were the small patient population and the extensive amount of SVF harvested from each method which included a substantial proportion of red blood cells. It should be noted that most of the techniques described in literature to obtain SVF are regulated by centrifugation, filtration and rinsing to avoid red blood cell contamination. We specifically did not undergo these procedures to analyze the entire SVF obtained from devices that may be used as infiltrative therapy for the treatment of OA in a single-step procedure (Pak et al., 2018).

Enzymatic methods have shown to be much more effective in terms of SVF cell recovery. Enzymatic methods employ proteolytic enzymes to disrupt the extracellular matrix which holds adipose tissue together. Collagenase-based enzymatic methods have reported nucleated cell yields much higher than mechanical methods. Furthermore, the composition of the cell population recovered by mechanical methods has a greater frequency of peripheral blood mononuclear cells with lower frequency of progenitor cells. While enzymatic methods yield consistently higher progenitor cells, non-enzymatic methods are not without merit. The differences resulting in the yields using mechanical and enzymatic methods can be partially attributed to the physical location of SVF cells in adipose tissue in perivascular space (niche). Mechanical methods of SVF isolation do not afford the same release of cells from the perivascular spaces because the disruption of the extracellular matrix is significantly reduced compared to enzymatic methods. As a result, the composition of the SVF resulting from mechanical isolations tends to be deficient in CD34 expression, with longer culture times. This issue is reflected much on the ability to differentiate into typical lineage. Only enzymatic technique showed good differentiation at proper time, instead we did not obtain differentiation from both mechanical devices, exception from ability to chondrocytic differentiation for Rigenera^®^. We explain this for the slower growth and the difficulty in reaching confluence but also because mechanical devices are able to select pre-commitment cells, that have not potentiality to differentiate in all cellular lineage of mesodermal leaflet, may because they have already lost CD34 positivity.

Literature has extensively reported SVF as a promising tool in orthopedics (Striano et al., 2015; Franceschini et al., 2016; Usuelli et al., 2018) but it is noteworthy to consider in our opinion, the immune-modulation, anti-inflammatory, and homeostatic properties of SVF within the joint through growth factors and cytokines, and not the ability of ASCs to regenerate cartilage (Maumus et al., 2013; Stromps et al., 2014). However, it would be interesting to better investigate whether ASCs injected within the joint may migrate to the cartilage lesions and promote healing and regeneration. Moreover, SVF is accessible and does not require cell separation or culturing conditions. Thus, the therapeutic cellular product is instantaneously obtained and has minimal contact with reagents resulting in a safer and more eligible option concerning regulatory standards. In the light of current knowledge, we hypothesize that the amount of ASCs obtained from the SVF is irrelevant to the mechanical procedure in clinical settings due to insufficient data and in relation to the minimum number of cells and the clinical improvement. In conclusion, investigations on adipose tissue-derived cell isolation systems have been conducted to ensure a safe and sterile isolation technique without contamination and unpredictability of cell material. Every method or system, clearly holds specific advantages and disadvantages in cost and handling. For these reasons, additional preclinical and clinical studies are required to support these preliminary findings.

## Conclusion

Mechanical techniques are effective in isolating SVF from adipose tissue with low differentiative capability but high immunomodulatory effect through cytokines and growth factors release. Mechanical methods are appealing because they are simple, quick and generally not associated with expensive equipment or disposables. While more expensive, enzymatic methods obtained more nucleated cells with a higher number of progenitor cells per volume of lipoaspirate processed with high ability of differentiation. Moreover, the FDA very clearly states that the isolation of SVF cells from adipose tissues, with or without the use of proteolytic enzymes, results in a final product which is considered to be “more than minimally manipulated” because the original structure of the adipose tissue has been significantly altered. All this is overtaken by mechanical systems that are capable of minimizing tissue manipulation. To date, the research must be oriented toward mechanical systems that are able to isolate CD34+ progenitor cells with the same capacity as the cells obtained from enzymatic digestion. To date, instead, the mechanical systems are able to provide a good cocktail of growth factors with an immunomodulatory activity for palliative-type clinical uses.

## Ethics Statement

The study respects all ethical requirements in its objectives and methodologies. We strictly comply with widely recognized international codes of practice such as the Nuremberg code, the Helsinki agreement, the conventions of the Council of Europe on human rights and biomedicine, with particularly attention to EU legislation: 2001/83/EC, 86/609/EEC, and FP7 Decision no. 1982/2006EC. Human biological samples are necessary because we need to test human cells, which have unique biological characteristics, distinct from those of animals. The overall intention in the project is to reduce the number of animal experiments. Only adult patients who are able to give consent will be included. All the patients, that are the subjects of our study, donated their consensus to scientific treatment and publication of their clinic situation and images. We have obtained written informed consent from all patients. This study was approved by our Internal Ethical Committee without any registration in public registry because this study is not a clinical trial.

## Author Contributions

LS, AG, MR, and FDF conceived of the present work. LS, FDF, and MR harvested the samples for the experiments. SM, GP, and GG performed the experiments. LS, FDF, and LF wrote the manuscript. FDF, AG, and MR supervised the findings of this work and helped to supervise the project. All authors made substantial intellectual contributions to the evolution of the concepts presented in the manuscript and approved the final version submitted.

## Conflict of Interest Statement

The authors declare that the research was conducted in the absence of any commercial or financial relationships that could be construed as a potential conflict of interest.
